# Nomogram model for predicting incomplete immune reconstitution in people living with HIV based on clinical characteristics

**DOI:** 10.3389/fimmu.2026.1762071

**Published:** 2026-03-02

**Authors:** Yaqiong Zhang, Wenjia Hu, Xiaoxia Zhang, Yulin Zhang, Zhongwei Zhang, Qunqun Jiang, Shan Wang, Yong Nan, Shihui Song, Yong Xiong

**Affiliations:** 1 Department of Infectious Diseases, Zhongnan Hospital of Wuhan University, Wuhan, China; 2 Center for AIDS Research, Wuhan University, Wuhan, China; 3 Department of Infectious Diseases, Xishui County People’s Hospital, Xishui, China

**Keywords:** antiretroviral therapy, human immunodeficiency virus, immune non-response, nomogram, prediction model

## Abstract

**Background:**

Despite the success of antiretroviral therapy (ART) for human immunodeficiency virus (HIV) in China, immune non-response (INR) remains critical for the long-term quality of life of people living with HIV (PLWH). Although the consensus on diagnosis and management of immunological non-responders in HIV infection (Version 2023) was published in China to standardize diagnostic criteria, prediction models for INR based on these criteria remain scarce. This study aims to develop and validate a nomogram model for early identification of INR risk based on the diagnostic criteria in this consensus, so as to facilitate clinical intervention.

**Methods:**

In this retrospective study, the primary cohort included 615 PLWH who initiated ART and completed over 4 years of follow-up at Zhongnan Hospital of Wuhan University (January 2016 to May 2025). They were randomly split into a training set (n=433) and an internal validation set (n=182) in a 7:3 ratio. An external validation set comprised 213 PLWH from Xishui County People’s Hospital (January 2012 to August 2025). Least absolute shrinkage and selection operator (LASSO) and multivariable logistic regression were used to identify independent predictors of INR, and a nomogram was constructed. The receiver operating characteristic (ROC) curves, calibration curves, and decision curve analysis (DCA) were employed to evaluate the discrimination, calibration, and clinical utility of the model in the training set and validation sets, respectively.

**Results:**

LASSO regression identified seven candidate variables: age at ART initiation, baseline CD4^+^ T cell count, body mass index (BMI), white blood cell count (WBC), hemoglobin (Hb), aspartate aminotransferase (AST), and World Health Organization (WHO) clinical stage. Subsequent multivariable logistic regression confirmed baseline CD4^+^ T cell count (p < 0.001), age at ART initiation (p = 0.001), and AST level (p = 0.014) as independent INR predictors. The resulting nomogram demonstrated area under the curve (AUC) values of 0.896, 0.903, and 0.766 in the training, internal validation, and external validation sets, respectively. The calibration curve and DCA further indicated satisfactory consistency and clinical net benefit.

**Conclusion:**

The developed nomogram effectively predicts INR risk in PLWH initiating ART, providing clinicians a practical tool for individualized management to improve patient prognosis.

## 1 Introduction

According to the latest report from the China Center for Disease Control and Prevention, as of December 31, 2024, there were 1,355,017 people living with human immunodeficiency virus (HIV)/acquired immunodeficiency syndrome (AIDS) in China, with 491,437 cumulative reported deaths (1). Current first-line antiretroviral therapy (ART) in China, typically consisting of nucleoside reverse transcriptase inhibitors (NRTIs) combined with non-nucleoside reverse transcriptase inhibitors (NNRTIs), protease inhibitors (PIs), or integrase strand transfer inhibitors (INSTIs), effectively suppresses viral replication (2). With the widespread application of ART, the viral load suppression rate among people living with HIV (PLWH) in China had risen to 95.4% by the end of 2021 (3). However, despite the significant overall effectiveness of AIDS antiviral therapy and the continuous decline in the population mortality rate, there remains a subset of patients who, despite achieving virological suppression (viral load < 50 copies/mL), experience difficulty in restoring their CD4^+^ T lymphocyte counts to a relatively satisfactory immune function level (≥350 cells/μL). This population is clinically defined as immunological non-responders (INRs) (4). INRs face a substantially higher risk of mortality and opportunistic infections compared to immunological responders (IRs), with non-AIDS-defining events becoming their leading cause of death, severely impacting long-term quality of life (5, 6).

In recent years, immune non-response (INR) has attracted increasing clinical attention, and its occurrence is closely associated with multiple risk factors. Previous studies have shown that advanced age and low pre-treatment CD4^+^ T cell count are major risk factors for INR (7–10). Other reported factors include the route of infection (11), ART regimen (12–14), delayed ART initiation (15), and World Health Organization (WHO) clinical stage (16) are also associated with poor prognosis in PLWH; however, the specific roles of these variables in immune reconstitution assessment remain controversial. Various laboratory parameters, such as white blood cell count (WBC) (17), platelet count (PLT) (18, 19), hemoglobin (Hb) (20, 21), alanine aminotransferase (ALT) (22), aspartate aminotransferase (AST) (16), and serum creatinine (SCr) (23), have also been linked to HIV disease progression and mortality. Furthermore, viral coinfections (e.g., hepatitis B and C) have been confirmed to impede immune recovery (24–26). The pathogenesis of INR has not been fully elucidated. Existing studies suggest that it is associated with an imbalance between T lymphocyte production and apoptosis caused by insufficient thymic output (27, 28), chronic immune activation and T-cell exhaustion (29, 30), intestinal flora disturbance and microbial translocation (31), as well as metabolic abnormalities such as ferroptosis and mitochondrial dysfunction in CD4^+^ T cells (32).

Following a systematic review of the existing literature on prediction models for INR in PLWH, we identified several limitations in current research. First, there is a lack of uniformity in the definition and diagnostic criteria for INR across different studies. This leads to heterogeneity in the outcome variables targeted by the prediction models, making direct comparison and integration of findings difficult and consequently limiting their clinical relevance (17). Second, regarding model development, existing studies have predominantly focused on predicting the risk of early INR after treatment initiation. For instance, although the study by Zhang et al. (16) incorporated a broad set of variables to enhance predictive comprehensiveness, some indicators (e.g., specific infection history) are not routinely available in clinical practice, which may compromise the model’s utility. Furthermore, the prediction time window of this model is primarily confined to the initial treatment phase (e.g., 2 years after treatment). Key performance metrics, such as discrimination and calibration, have not been adequately validated in patients receiving long-term virologically suppressive therapy. Therefore, its applicability for guiding INR management in individuals on extended treatment (e.g., beyond 4 years) remains limited.

Although China issued the consensus on diagnosis and management of immunological non-responders in HIV infection (Version 2023) in recent years to standardize diagnostic criteria (4), prediction models based on this consensus remain scarce. To fill this research gap, this study strictly adheres to the above consensus standards to develop and validate a risk prediction model for INR.

Machine learning algorithms have shown significant promise in disease diagnosis and prognosis prediction (33). As an intuitive risk visualization tool, the nomogram can integrate multiple predictive variables into an evaluation model for the probability of clinical events based on the results of multivariable regression analysis. Therefore, to improve the accuracy and clinical utility of INR risk identification, this study combines machine learning-based variable selection with nomogram model construction, aiming to provide an effective tool for the early identification of high-risk INR individuals for targeted intervention.

## 2 Methods

### 2.1 Study population and design

**Figure 1:**
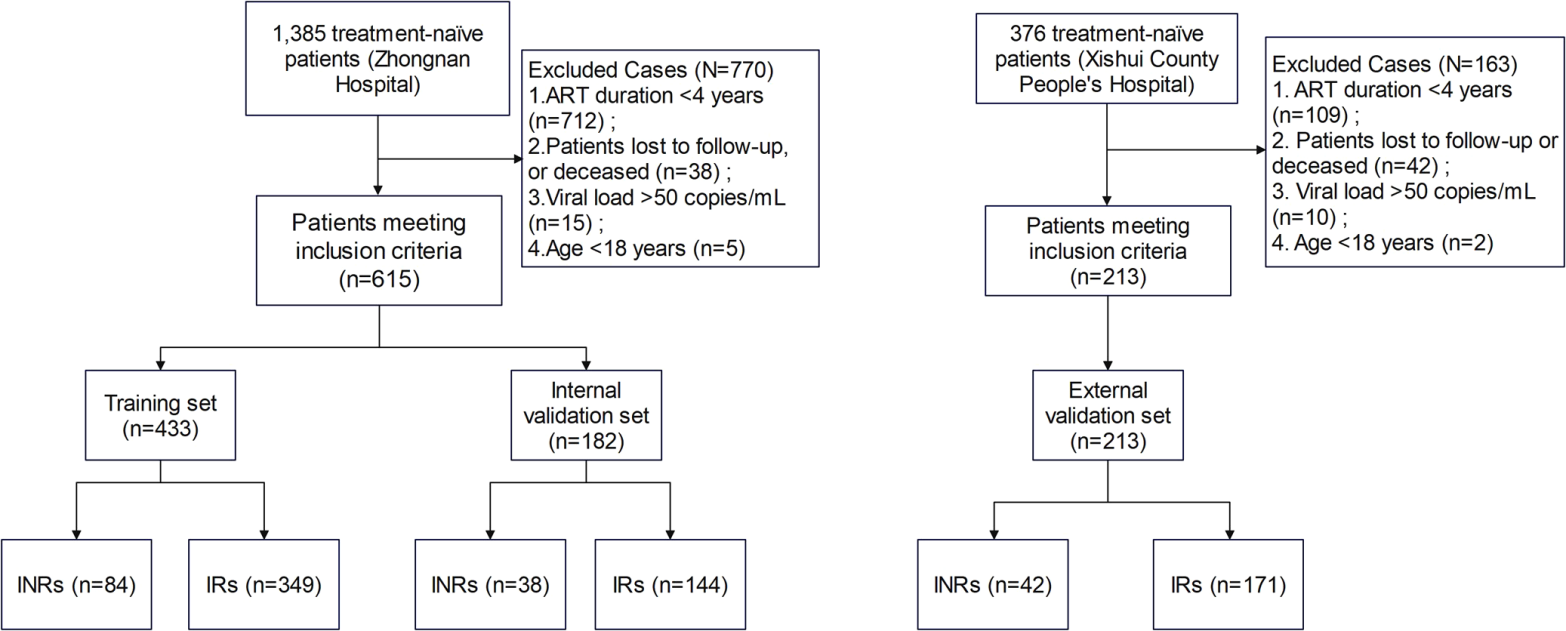
Flow diagram of screening and enrollment of study participants.

This study is a retrospective cohort study. The study cohort and flowchart are presented in **Figure 1**. The primary study subjects were derived from PLWH who initiated ART and were followed up at Zhongnan Hospital of Wuhan University between January 2016 and May 2025, serving as the development cohort. A total of 1385 cases were initially screened. The inclusion criteria were as follows: 1) HIV infection diagnosis conforming to the Chinese guidelines for the diagnosis and treatment of HIV/AIDS (2024 edition) (2); 2) Receipt of standardized ART for over 4 years; 3) Sustained HIV viral load (VL) < 50 copies/mL for 3 consecutive years; 4) Age ≥ 18 years. The exclusion criteria included: 1) Loss to follow-up or death during the study period; 2) Presence of other immunosuppressive conditions (e.g., hematological malignancies, organ transplantation, long-term immunosuppressant use, severe renal insufficiency, or liver cirrhosis); 3) Missing key follow-up laboratory data (e.g., CD4^+^ T cell count); 4) Pregnancy or lactation. Based on the above criteria, 615 patients were ultimately included. For model development and internal validation, this cohort was randomly divided into a training set (n=433) and an internal validation set (n=182) in a 7:3 ratio.

Subsequently, to evaluate the generalizability of the model, an independent cohort from Xishui County People’s Hospital was included. This cohort consisted of PLWH who initiated ART and were followed up between January 2012 and August 2025, with 376 cases initially screened. After applying the same inclusion and exclusion criteria as those used for the development cohort, 213 patients constituted the external validation cohort.

In this study, INR was defined per the 2023 consensus (4). PLWH who received standardized ART for more than 4 years, had a viral load confirmed to be < 50 copies/mL by high-sensitivity assay for 3 consecutive years, and maintained a CD4^+^ T cell count below 350 cells/μL during annual follow-up were defined as INRs. Correspondingly, IRs were patients with a CD4^+^ T cell count ≥ 350 cells/μL under the identical treatment and virological suppression conditions, serving as the control group in this study. The study was reviewed and approved by the Medical Ethics Committee of Zhongnan Hospital of Wuhan University and Xishui County People’s Hospital.

### 2.2 Data collection of candidate variables

Two trained physicians extracted data from electronic medical records using standardized Excel forms. Demographic variables included gender and age at ART initiation. Disease-related characteristics encompassed route of infection, ART regimen, WHO clinical staging, duration of delay in ART initiation, and comorbid infections. Comorbid infections were defined as positivity for hepatitis B surface antigen (HBsAg) or hepatitis C virus antibody (anti-HCV) at the time of HIV diagnosis. Laboratory and physical examination parameters (with normal reference ranges in parentheses) included: CD4^+^ T lymphocyte count, HIV VL (lower limit of detection < 20 copies per milliliter [cps/mL]), WBC (4–10 × 10^9^/L), PLT (100–300 × 10^9^/L), Hb (120–160 g/L), SCr, triglycerides (TG; < 1.7 mmol/L), total cholesterol (TC; < 5.18 mmol/L), ALT (9–50 U/L), AST (15–40 U/L), and body mass index (BMI) (18.5–23.9 kg/m^2^). All laboratory assays were performed by trained technicians in the hospital’s clinical laboratory, in strict compliance with established clinical guidelines.

### 2.3 Statistical analysis

All data analyses in this study were performed using SPSS (version 27.0) and R software (version 4.3.3). First, the extent of missing data was assessed for all variables included in the analysis. The missing rates for baseline laboratory variables (including WBC, PLT, Hb, SCr, TG, TC, AST, and ALT) were all below 5%. To evaluate the missing data mechanism, we compared the distribution of key baseline characteristics between patients with missing data and those with complete data. The results indicated no significant systematic differences between the groups (all p > 0.05), supporting the assumption that the data were missing at random, as detailed in the **Supplementary Material**. Given the low proportion of missingness, multiple imputation was employed to handle the missing data.

Categorical variables are presented as frequencies (percentages), while continuous variables with non-normal distributions are described as medians (interquartile ranges, IQRs). Comparisons of categorical variables between groups were conducted using the Chi-square test or Fisher’s exact test, and comparisons of continuous variables were performed using the Mann-Whitney U test.

First, least absolute shrinkage and selection operator (LASSO) regression was employed to screen for potential predictors of INR in HIV/AIDS patients. The analysis was conducted using the “glmnet” package in R language. This method introduces L1 regularization to penalize the absolute values of regression coefficients, shrinking coefficients of weakly correlated variables to zero, thereby achieving variable selection and model simplification while effectively mitigating multicollinearity. The optimal tuning parameter λ (lambda) was determined through ten-fold cross-validation to balance model complexity and goodness-of-fit, and predictor variables with non-zero coefficients were selected. Subsequently, the variables identified by LASSO regression were incorporated into a multivariable logistic regression model to ultimately determine the independent influencing factors of INR. Based on the above factors, a prognostic nomogram was constructed using the “rms” package, transforming the complex regression model into a visual clinical tool to facilitate individualized risk assessment.

The model’s performance was evaluated in the training and validation sets. The “pROC” package was employed to plot receiver operating characteristic (ROC) curves and calculate the area under the curve (AUC), assessing the model’s discriminative ability. Calibration curves were plotted using the “rms” package to examine the agreement between predicted probabilities and actual probabilities. Decision curve analysis (DCA) was performed using the “rmda” package to evaluate the model’s clinical net benefit. A two-tailed P value < 0.05 was considered statistically significant for all statistical analyses.

## 3 Results

### 3.1 Baseline characteristics of the study population

To assess the randomness of the missing data, a pooled analysis was conducted on all cases with missing laboratory indicators across both cohorts. The results showed that, compared to patients with complete data, those missing at least one laboratory value demonstrated no statistically significant differences in key baseline characteristics, including age at ART initiation, baseline CD4^+^ T-cell count, BMI, sex, WHO stage, ART regimen, and route of infection (all P > 0.05). This indicates that the data were missing at random, with no substantial selection bias introduced. The missing data were handled using the multiple imputation method described previously, and all subsequent analyses were performed on the imputed complete datasets.

Based on the predefined inclusion and exclusion criteria, a total of 615 patients were ultimately enrolled in the entire development cohort. INRs accounted for 19.84% (122/615). ART for all patients was initiated before May 2021, with a median follow-up time of 5 years (IQR: 4–5 years). For predictive model construction, the development cohort was randomly divided into a training set (n=433) and an internal validation set (n=182) in a 7:3 ratio. The proportions of INRs in the training and validation sets were 19.40% (84/433) and 20.88% (38/182), respectively. The external validation cohort ultimately included 213 patients (all of whom initiated ART before August 2021), among whom INRs accounted for 19.72% (42/213), with a median follow-up time of 8 years (IQR: 6–10 years).

Overall, 828 patients were included in the study. Comparisons between the internal validation set and the training set revealed no statistically significant differences in the observed indicators (all p > 0.05). In contrast, comparisons between the external validation set and the training set showed statistically significant differences in indicators such as sex, route of infection, ART regimen, baseline CD4^+^ T cell count, WHO stage, number of abnormal cases of WBC, PLT, Hb, and SCr, ALT, AST, age at ART initiation, and delay in ART initiation (p < 0.05). The baseline clinical characteristics are detailed in **Table 1**.

**Table 1:** Baseline characteristics of PLWH.

Variable	Training set (N = 433)	Internal validation set (N = 182)	External validation set (N = 213)	P1-value	P2-value
Sex (%)				0.454^a^	< 0.001^a^
Female	22(5.08%)	12(6.59%)	56(26.29%)		
Male	411(94.92%)	170(93.41%)	157(73.71%)		
Route of Infection (%)				0.445^b^	< 0.001^a^
Heterosexual Transmission	122(28.18%)	43(23.63%)	150(70.42%)		
Homosexual Transmission	306(70.67%)	138(75.82%)	44(20.66%)		
Others	5(1.15%)	1(0.55%)	19(8.92%)		
ART Regimen (%)				0.555^b^	< 0.001^b^
NRTI+NNRTI	358(82.68%)	145(79.67%)	210(98.59%)		
NRTI+INSTI	68(15.70%)	35(19.23%)	3(1.41%)		
Others	7(1.62%)	2(1.10%)	0(0.00%)		
HBsAg (%)				0.688^a^	0.628^a^
Negative	403(93.07%)	171(93.96%)	196(92.02%)		
Positive	30(6.93%)	11(6.04%)	17(7.98%)		
Anti-HCV (%)				0.636^b^	0.066^b^
Negative	430(99.31%)	180(98.90%)	207(97.18%)		
Positive	3(0.69%)	2(1.10%)	6(2.82%)		
Baseline CD4^+^ T Cell Count (%)				0.785^a^	< 0.001^a^
<200	134(30.95%)	63(34.62%)	110(51.64%)		
200~349	139(32.10%)	56(30.77%)	71(33.33%)		
350~499	105(24.25%)	39(21.43%)	22(10.33%)		
≥500	55(12.70%)	24(13.19%)	10(4.69%)		
BMI (%)				0.416^a^	0.069^a^
18.5~23.9	278(64.20%)	125(68.68%)	142(66.67%)		
<18.5	48(11.09%)	21(11.54%)	33(15.49%)		
>23.9	107(24.71%)	36(19.78%)	38(17.84%)		
WHO Clinical Stage (%)				0.157^a^	0.010^a^
I-II	234(54.04%)	87(47.80%)	92(43.19%)		
III-IV	199(45.96%)	95(52.20%)	121(56.81%)		
WBC (%)				0.491^a^	0.003^a^
≥4	339(78.29%)	147(80.77%)	144(67.61%)		
<4.0	94(21.71%)	35(19.23%)	69(32.39%)		
PLT (%)				0.859^a^	< 0.001^a^
≥100	415(95.84%)	175(96.15%)	182(85.45%)		
<100	18(4.16%)	7(3.85%)	31(14.55%)		
Hb (%)				0.712^a^	< 0.001^a^
≥120	371(85.68%)	158(86.81%)	145(68.08%)		
<120	62(14.32%)	24(13.19%)	68(31.92%)		
Abnormal SCr (%)				0.728^b^	< 0.001^a^
No	427(98.61%)	179(98.35%)	198(92.96%)		
Yes	6(1.39%)	3(1.65%)	15(7.04%)		
TG (%)				0.949^a^	0.136^a^
<1.70	327(75.52%)	137(75.27%)	172(80.75%)		
≥1.70	106(24.48%)	45(24.73%)	41(19.25%)		
TC (%)				0.466^a^	0.367^a^
<5.18	398(91.92%)	164(90.11%)	200(93.90%)		
≥5.18	35(8.08%)	18(9.89%)	13(6.10%)		
ALT (%)				0.761^a^	0.010^a^
≤50	367(84.76%)	156(85.71%)	196(92.02%)		
>50	66(15.24%)	26(14.29%)	17(7.98%)		
AST (%)				0.761^a^	0.045^a^
≤40	367(84.76%)	156(85.71%)	167(78.40%)		
>40	66(15.24%)	26(14.29%)	46(21.60%)		
Age at ART Initiation (years)	29.00(24.00,39.50)	29.00(23.00,42.00)	45.00(35.00,54.00)	0.816	< 0.001
Delay in ART Initiation (days)	27.00(16.00,56.00)	23.00(15.75,56.25)	31.00(15.00,176.00)	0.751	0.034
Baseline HIV VL (lg (x+1))	4.46(3.63,4.99)	4.52(3.89,5.00)	–	0.346	–

P1-value represent the results of comparisons between the training set and internal validation set, used to assess the balance of baseline data between the two groups; P2-value are for evaluating differences in baseline characteristics between the training set and external validation set.

a Pearson’s chi-square test.

b Fisher’s exact test. “Other” in ART regimens includes protease inhibitors (PI) and other classes of antiviral drugs. Abnormal SCr is defined as SCr > 104 μmol/L in males and > 84 μmol/L in females. Baseline HIV VL data were transformed using lg(x+1), with the formula: y = lg(VL + 1). Due to the lack of specific requirements for HIV VL testing in primary hospitals at the time of patient enrollment, the baseline values of this indicator had a high missing rate in the data from Xishui County People’s Hospital; therefore, it was not included in the external validation set analysis.

In the training set, comparisons of baseline characteristics between groups showed that route of infection (p = 0.017), HBsAg positivity (p = 0.003), baseline CD4^+^ T cell count (p < 0.001), BMI (p = 0.023), WHO clinical stage (p < 0.001), WBC (p < 0.001), PLT (p < 0.001), Hb (p < 0.001), ALT (p = 0.006), AST (p < 0.001), age at ART initiation (p < 0.001), and baseline HIV VL (p < 0.001) were statistically significant between the two groups (all p < 0.05). (**Table 2**).

**Table 2:** Baseline clinical characteristics of IR and INR in the training set.

Variable	IR group (N = 349)	INR group (N = 84)	Statistic	P-value
Sex (%)			χ^2^ = 0.164	0.685^a^
Female	17(4.87%)	5(5.95%)		
Male	332(95.13%)	79(94.05%)		
Route of Infection (%)			χ^2^ = 7.750	0.017^b^
Heterosexual Transmission	88(25.21%)	34(40.48%)		
Homosexual Transmission	257(73.64%)	49(58.33%)		
Others	4(1.15%)	1(1.19%)		
ART Regimen (n,%)			χ^2^ = 2.478	0.304^b^
NRTI+NNRTI	293(83.95%)	65(77.38%)		
NRTI+INSTI	51(14.61%)	17(20.24%)		
Others	5(1.43%)	2(2.38%)		
HBsAg (%)			χ^2^ = 8.748	0.003^a^
Negative	331(94.84%)	72(85.71%)		
Positive	18(5.16%)	12(14.29%)		
Anti-HCV (%)			–	0.477^b^
Negative	347(99.43%)	83(98.81%)		
Positive	2(0.57%)	1(1.19%)		
Baseline CD4^+^ T Cell Count (%)			χ^2^ = 136.515	< 0.001^a^
<200	64(18.34%)	70(83.33%)		
200~349	127(36.39%)	12(14.29%)		
350~499	103(29.51%)	2(2.38%)		
≥500	55(15.76%)	0(0.00%)		
BMI (%)			χ^2^ = 7.568	0.023^a^
18.5~23.9	216(61.89%)	62(73.81%)		
<18.5	37(10.60%)	11(13.10%)		
>23.9	96(27.51%)	11(13.10%)		
WHO Clinical Stage (%)			χ^2^ = 58.615	< 0.001^a^
I-II	220(63.04%)	14(16.67%)		
III-IV	129(36.96%)	70(83.33%)		
WBC (%)			χ^2^ = 45.034	< 0.001^a^
≥4	296(84.81%)	43(51.19%)		
<4.0	53(15.19%)	41(48.81%)		
PLT (%)			χ^2^ = 20.898	< 0.001^a^
≥100	342(97.99%)	73(86.90%)		
<100	7(2.01%)	11(13.10%)		
Hb (%)			χ^2^ = 43.334	< 0.001^a^
≥120	318(91.12%)	53(63.10%)		
<120	31(8.88%)	31(36.90%)		
Abnormal SCr (%)			–	0.330^b^
No	345(98.85%)	82(97.62%)		
Yes	4(1.15%)	2(2.38%)		
TG (%)			χ^2^ = 0.943	0.331^a^
<1.70	267(76.50%)	60(71.43%)		
≥1.70	82(23.50%)	24(28.57%)		
TC (%)			χ^2^ = 0.009	0.925^a^
<5.18	321(91.98%)	77(91.67%)		
≥5.18	28(8.02%)	7(8.33%)		
ALT (%)			χ^2^ = 7.680	0.006^a^
≤50	304(87.11%)	63(75.00%)		
>50	45(12.89%)	21(25.00%)		
AST (%)			χ^2^ = 19.909	< 0.001^a^
≤40	309(88.54%)	58(69.05%)		
>40	40(11.46%)	26(30.95%)		
Age at ART Initiation (years)	27.00(23.00,35.00)	37.00(29.00,50.00)	Z=6.060	< 0.001
Delay in ART Initiation (days)	27.00(16.00,56.00)	26.50(15.25,55.50)	Z=-0.123	0.902
Baseline HIV VL (lg (x+1))	4.37(3.45,4.92)	4.95(4.28,5.50)	Z=5.050	< 0.001

a Pearson’s chi-square test.

b Fisher’s exact test.

### 3.2 Screening and identification of predictive factors

**Figure 2:**
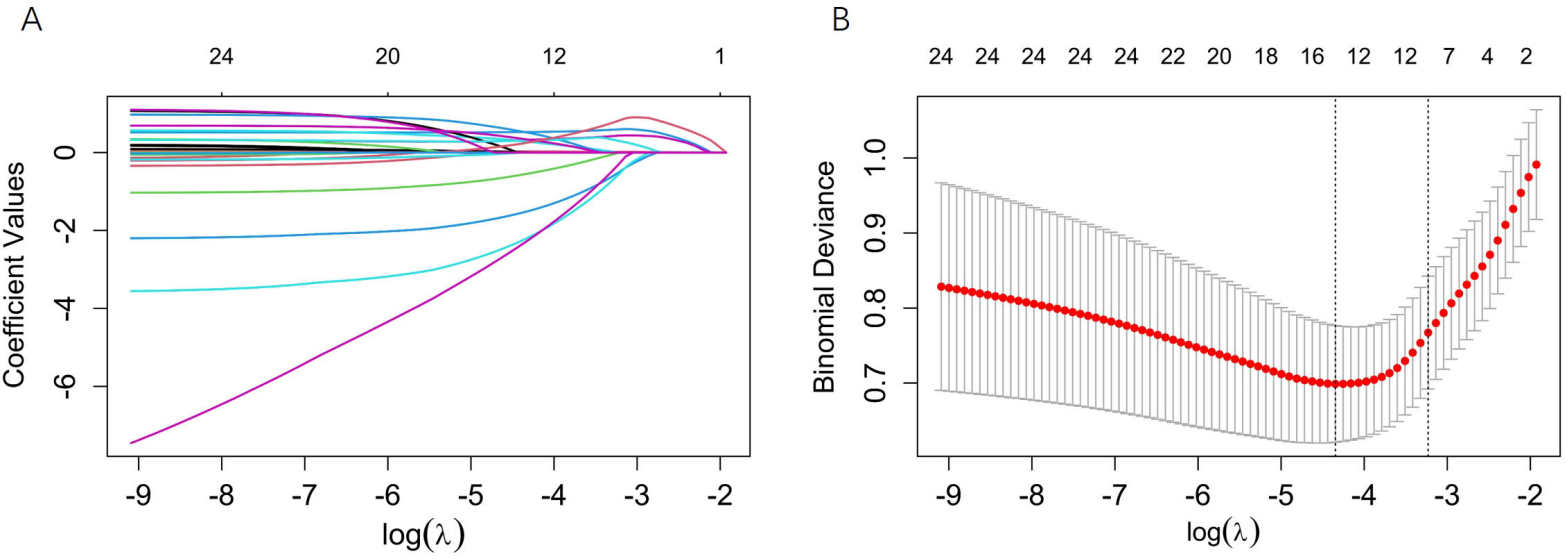
Clinical feature selection using LASSO regression. **(A)** (Left panel) LASSO coefficient path plot; **(B)** (Right panel) Ten-fold cross-validation curve plot.

In the training set, we employed LASSO regression for initial variable screening, incorporating a total of 19 potential predictor variables. The optimal regularization parameter was determined via ten-fold cross-validation, and λ.1se (λ = 0.040) was selected to obtain a more parsimonious model, resulting in the identification of 7 variables: age at ART initiation, baseline CD4^+^ T cell count, BMI, WBC, Hb, AST, and WHO clinical stage (**Figures 2A, B**). Subsequently, all these variables were incorporated into a multivariable logistic regression model for further screening and confirmation.

The final results indicated that baseline CD4^+^ T cell count (p < 0.001) was identified as a protective factor against INR in PLWH. Conversely, older age at ART initiation (p = 0.001) and elevated AST levels (> 40 U/L, p = 0.014) were determined to be independent risk factors for INR in this population. Only variables with independent predictive significance (p < 0.05) were retained to construct the Nomogram. (**Table 3**).

**Table 3:** Multivariable logistic regression analysis of factors associated with INR in PLWH in the training set.

Variable	OR	95%CI	P-value
Age at ART Initiation (years)	1.038	1.015~1.062	0.001
Baseline CD4^+^ T Cell Count (/μL)			< 0.001
200~349	0.124	0.053~0.287	< 0.001
350~499	0.029	0.006~0.148	< 0.001
≥500	0.000	0.000	0.997
BMI(kg/m2)			0.119
<18.5	0.907	0.330~2.499	0.851
>23.9	0.418	0.182~0.958	0.039
WBC (<4.0) (10^9^/L)	1.792	0.925~3.471	0.084
Hb (<120) (g/L)	1.487	0.691~3.200	0.311
AST (>40) (U/L)	2.602	1.212~5.586	0.014
WHO Clinical Stage (III-IV)	0.885	0.368~2.124	0.784

### 3.3 Construction and validation of the nomogram prediction model

**Figure 3:**
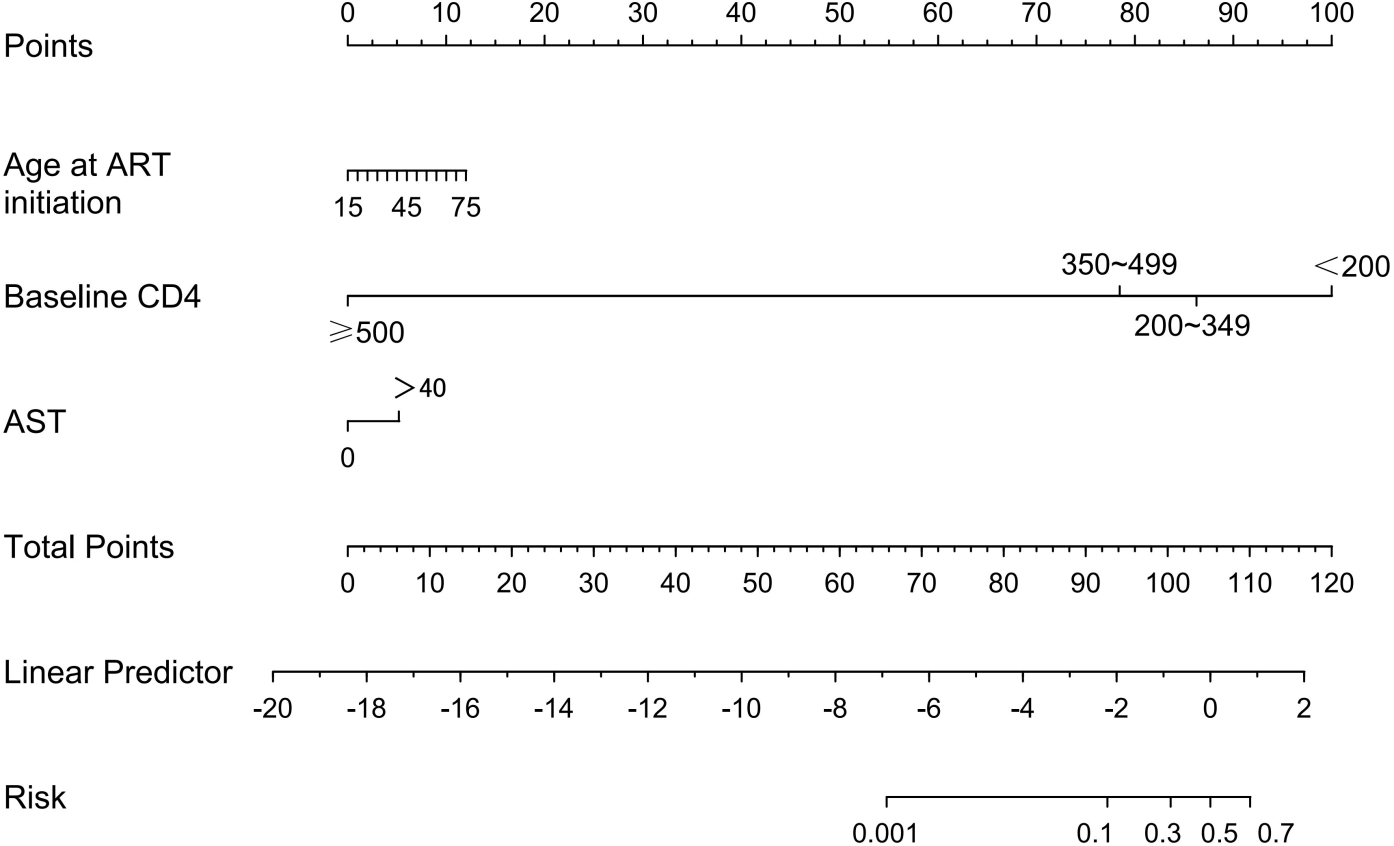
Nomogram for predicting INR in PLWH after ART initiation.

Based on the results of LASSO regression and multivariable logistic regression analyses, a nomogram prediction model incorporating age at ART initiation, baseline CD4^+^ T cell count, and AST level was constructed (**Figure 3**). Taking baseline CD4^+^ T cell count as an example: locate the corresponding point on the “Baseline CD4” axis, draw a vertical line upward to the “Points” axis to obtain the corresponding score; repeat this process for other variables to get their respective scores. Sum these scores, find the total on the “Total Points” axis, and draw a vertical line downward to the “Risk” axis to obtain the individual risk estimate. A higher total score indicates a higher risk of developing INR.

### 3.4 Evaluation of the nomogram model

**Figure 4:**
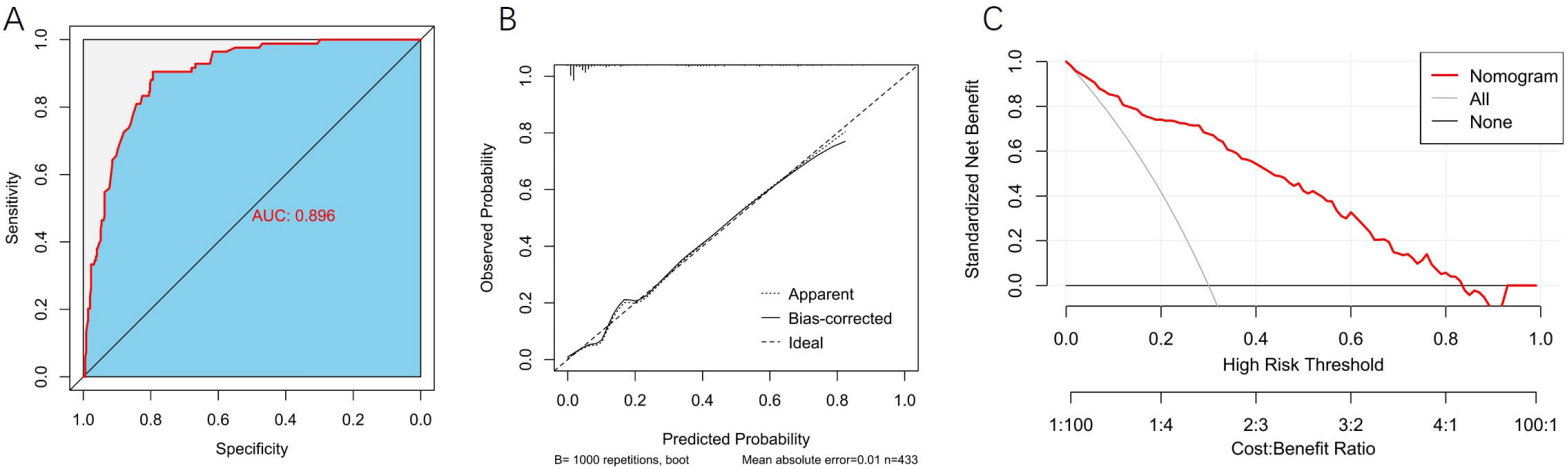
Evaluation of model performance in the training set. **(A)** ROC curve. **(B)** Calibration curve. **(C)** DCA.

In the training set, we constructed a ROC curve, a calibration curve, and a DCA to evaluate the model. Results showed that the area under the ROC curve (AUC) of the model in the training set was 0.896 (95% confidence interval [CI]: 0.863–0.929; **Figure 4A**), with a sensitivity of 90.48% and a specificity of 79.37%. After internal bootstrap validation (1000 repetitions), the mean absolute error of the calibration curve was 0.010, indicating good stability of the model (**Figure 4B**). DCA demonstrated that the model yielded a favorable clinical net benefit across the threshold probability range of 0.1 to 0.8 (**Figure 4C**).

**Figure 5:**
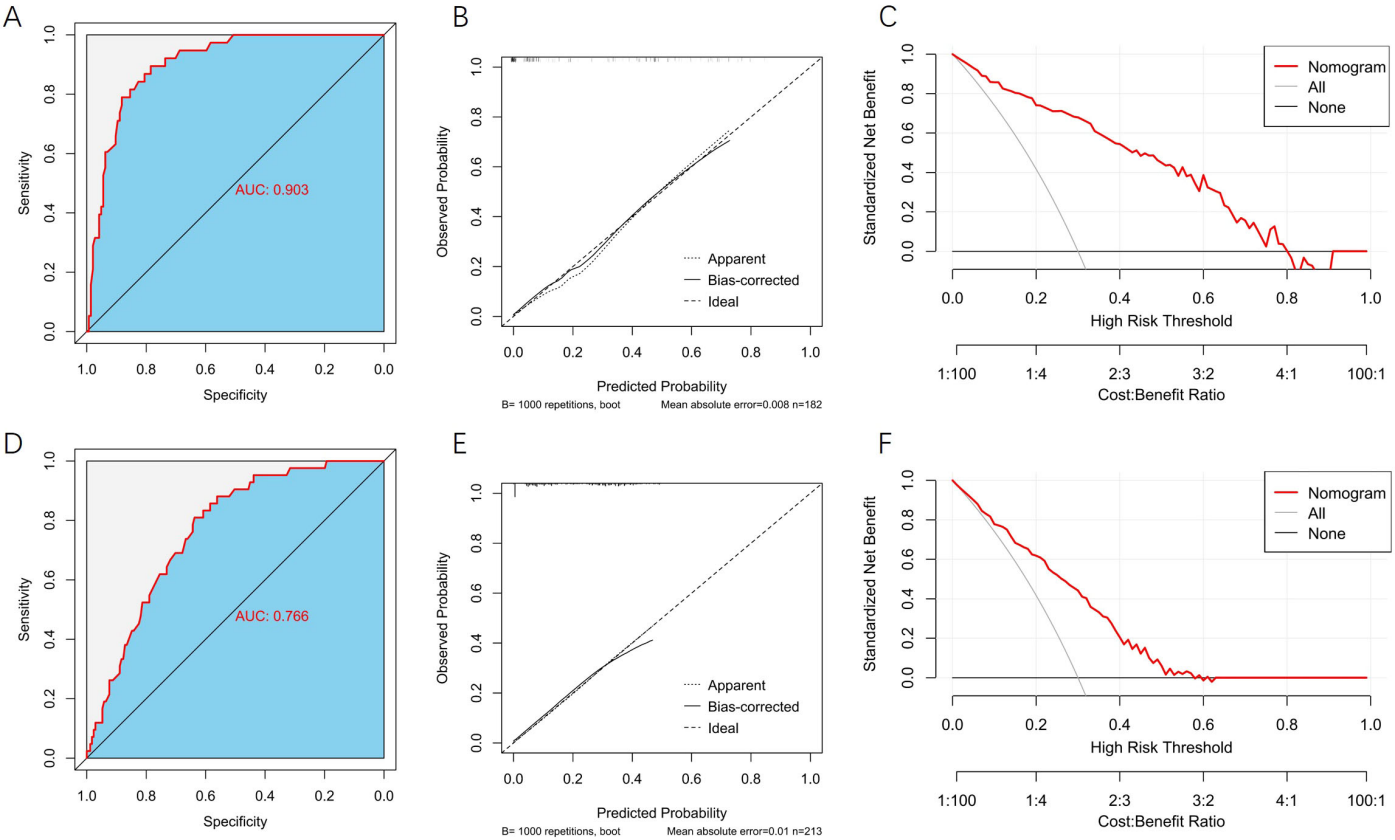
Evaluation of the nomogram prediction performance in validation sets. **(A)** ROC curve of the internal validation set. **(B)** Calibration curve of the internal validation set. **(C)** DCA of the internal validation set. **(D)** ROC curve of the external validation set. **(E)** Calibration curve of the external validation set. **(F)** DCA of the external validation set.

In the internal validation set, the model exhibited excellent discriminative ability, with an AUC of 0.903 (95% CI: 0.856–0.950; **Figure 5A**), a sensitivity of 89.47%, and a specificity of 78.47%. In the external validation set, the model yielded an AUC of 0.766 (95% CI: 0.695–0.837; **Figure 5D**), with a sensitivity of 80.95% and a specificity of 63.74%. Calibration curves demonstrated low mean absolute errors of 0.008 and 0.010 for the two validation sets, respectively (**Figures 5B, E**). DCA further confirmed that the predictive nomogram model provides substantial clinical net benefit (**Figures 5C, F**).

## 4 Discussion

The significantly elevated risk of adverse events in INRs creates an urgent clinical need for early identification of high-risk individuals. To address the long-standing core issue of inconsistent diagnostic criteria in this research area, this study strictly defined outcome events according to the latest Chinese expert consensus, aiming to provide more reliable evidence for this field.

This study identified age at ART initiation, pre-treatment CD4^+^ T cell count, and AST level as independent predictors of INR and constructed a predictive model based on these three factors, which is consistent with numerous previous reports (17, 21, 34). Existing studies have shown a positive correlation between age at ART initiation and INR risk (8, 35), while a lower pre-treatment CD4^+^ T cell count is a strong predictor, demonstrating a significant negative correlation with INR occurrence (36, 37). Further research by Li et al. revealed that the CD4^+^ T cell count in individuals over 50 years old was significantly lower than in those under 35. Although ART helped restore CD4^+^ T cell counts to some extent across all age groups, younger patients exhibited significantly superior immune reconstitution rates and degrees of recovery compared to older patients. Moreover, the inhibitory effect of age on CD4^+^ T cell recovery became increasingly pronounced with longer treatment duration (38). Mechanistically, the thymus, as the primary site for generating immunocompetent T cells, undergoes progressive functional decline with age, leading to restricted nascent T cell output and consequently impairing CD4^+^ T cell recovery (39). Additionally, elderly patients have shortened T cell telomere lengths, weakened proliferation ability, and are prone to immunosenescence (40). This mechanistic understanding supports the negative impact of advanced age on INR observed in our study. Research indicates that advanced age and low CD4^+^ T cell count are major risk factors for significant clinical events such as cardiovascular events and non-AIDS-defining malignancies (5). Another study reported that the median age of the high-risk group for atherosclerotic cardiovascular disease among PLWH was 66 years, significantly higher than the 52 years in the low-risk group (41), underscoring the critical role of age in non-AIDS-related complications. Furthermore, a study observing immune recovery after one year of ART noted that patients with a baseline CD4^+^ T cell count <50/μL struggled to achieve a CD4^+^ T cell count above 200/μL despite standardized ART (42). Another study also emphasized that the baseline CD4^+^ T cell count is a sensitive indicator for predicting immune reconstitution outcomes (43).

AST serves as a crucial biomarker for liver function assessment. Relevant studies indicate that AST levels are closely and positively correlated with viral suppression and immune recovery in PLWH (16). As a sensitive indicator of hepatocellular damage, elevated AST levels reflect hepatic inflammation or necrosis. Impaired liver function subsequently reduces the capacity to clear toxins and inflammatory factors, leading to a systemic chronic low-grade inflammatory state and persistent immune activation. This pathological environment can accelerate functional exhaustion of CD4^+^ T cells, thereby impeding the immune reconstitution process (44). In our study, patients with AST levels >40 U/L exhibited a 2.60-fold higher risk of developing INR compared to those with AST ≤40 U/L, further supporting the value of incorporating AST levels as a predictive indicator for INR occurrence.

This study addresses the clinically relevant issue of CD4^+^ T cell recovery plateau following long-term viral suppression within the context of China’s predominant ART regimens, which primarily consist of two NRTIs combined with either one NNRTI, one PI, or one INSTI (45). This focus underscores the study’s significant practical relevance and research necessity. By tracking annual CD4^+^ T cell count changes from treatment initiation until the follow-up cutoff, and applying the INR diagnostic criteria recommended by the world’s first expert consensus on INR, we conducted a retrospective analysis of pre-ART clinical data from 615 PLWH at a single tertiary hospital in a major central Chinese city. We identified age, CD4^+^ T cell count, and AST level as baseline risk factors for INR and pioneered the development of a predictive nomogram model based on these three parameters. External validation was performed using a follow-up cohort from a primary hospital.

The model demonstrated exceptional discrimination in both the training set (AUC = 0.896) and internal validation set (AUC = 0.903), indicating excellent predictive performance. Notably, in the independent external validation set, the model achieved an AUC of 0.766, exceeding the empirical threshold of 0.75, which suggests reasonable generalizability. Furthermore, calibration curves and decision curve analysis confirmed the model’s satisfactory calibration and clinical applicability. This study facilitates early risk stratification and tailored follow-up management for PLWH at high risk of INR, ultimately aiming to improve patient prognosis and quality of life.

Furthermore, compared to the relatively lenient criteria used in earlier studies (46), the updated standards provide a more precise definition of INR. Previous criteria often suffered from limitations such as short observation periods and inconsistent thresholds. In contrast, the new standards not only require a sustained CD4^+^ T-cell count below a specific threshold but also emphasize evaluation under the condition of prolonged viral suppression (e.g., >3 years). This approach more accurately identifies the true “inadequate immune reconstitution” population and reduces misclassification due to incomplete viral suppression or other confounding factors. Consequently, the predictors identified in this context—such as baseline CD4^+^ T-cell count, AST level, and age—carry greater biological relevance and robustness.

In terms of clinical interpretation, models based on the updated criteria place greater emphasis on identifying high-risk individuals. Older criteria may have included many patients with relatively low long-term risk, whereas the predicted probabilities from our model better reflect a patient’s actual risk of long-term adverse outcomes, thereby offering a more reliable basis for clinical decision-making.

More importantly, the evolution of diagnostic consensus reflects a deepened understanding of disease prognosis management. Previously, inconsistent diagnostic criteria often led to unclear indications and necessity for clinical intervention. The predictive model constructed according to the new standards enables risk stratification and facilitates the early identification of patients who, despite successful virological control, remain in an immunodeficient state and thus require closer monitoring or consideration of adjunctive immune interventions. For example, in patients classified as high-risk by the model, clinicians may consider shortening follow-up intervals, enhancing screening for opportunistic infections, or more actively exploring opportunities for participation in immunotherapy clinical trials.

Several limitations warrant consideration. First, a degradation in model performance was observed in the external validation cohort, as indicated by a decrease in the AUC values. The observed decline in performance in the external validation set may stem from significant demographic and clinical differences between the tertiary and primary hospital cohorts. Specifically, notable disparities were identified: the male-to-female ratio was approximately 3:1 in the external cohort compared to about 19:1 in the training cohort. Furthermore, heterosexual transmission was predominant in the external cohort, whereas homosexual transmission was more common in the training cohort. Patients in the external cohort also had a higher overall age at ART initiation. In addition, marked differences existed in baseline laboratory indicators (e.g., WBC, PLT, Hb) between the two cohorts.

These discrepancies may affect the model’s generalizability through several mechanisms. Firstly, baseline immune status and laboratory profiles likely exhibit population-specific variations across different genders, transmission routes, and age groups. Consequently, a model developed on the training cohort may naturally show reduced predictive accuracy when applied to an external population with a different feature distribution. Secondly, these phenotypic characteristics could act as proxies for deeper, unmeasured biological factors—such as viral subtypes, specific inflammatory biomarkers, or host genetic variations—that may influence INR, potentially explaining part of the performance gap (29, 47). Thirdly, since our analysis confirmed that age at ART initiation is an independent predictor of incomplete immune reconstitution, the difference in age distribution between cohorts could directly impact the model’s calibration and discriminative ability. Finally, the retrospective design is susceptible to selection and attrition bias.

Future studies should aim to enhance the model’s robustness by expanding the sample size through the inclusion of patient cohorts from more diverse geographic regions and ethnic backgrounds. The consideration of more advanced machine learning algorithms may also be warranted to better adapt the tool to various clinical scenarios.

In summary, we have constructed a practical nomogram that effectively predicts the risk of incomplete immune reconstitution in virologically suppressed PLWH. By integrating age at ART initiation, baseline CD4^+^ T cell count, and AST level, this tool allows for early risk stratification. It can assist clinicians in identifying high-risk INR individuals who may benefit from more intensive monitoring, investigation of comorbidities, and personalized management strategies, ultimately aiming to improve long-term clinical outcomes.

## Data Availability

The original contributions presented in the study are included in the article/**Supplementary Material**. Further inquiries can be directed to the corresponding authors.
