# Supplementary material for: Nomogram model for predicting incomplete immune reconstitution in people living with HIV based on clinical characteristics
Source: Front Immunol. 2026 Mar 2;17:1762071. doi: 10.3389/fimmu.2026.1762071 (PMC12989480; doi:10.3389/fimmu.2026.1762071)
Supplement: Supplementary file 1 [file Table1.docx]

|  | Zhongnan Hospital of Wuhan University | | Xishui County People's Hospital | | |
| --- | --- | --- | --- | --- | --- |
| Variable | Missing cases (n) | Missing (%) | Missing cases (n) | | Missing (%) |
| WBC | 1 | 0.16 | 1 | 0.47 | |
| PLT | 1 | 0.16 | 2 | 0.94 | |
| Hb | 1 | 0.16 | 1 | 0.47 | |
| SCr | 2 | 0.33 | 8 | 3.76 | |
| TG | 16 | 2.60 | 10 | 4.69 | |
| TC | 17 | 2.76 | 9 | 4.23 | |
| AST | 1 | 0.16 | 1 | 0.47 | |
| ALT | 2 | 0.33 | 1 | 0.47 | |

Table 1. Missing Data Statistics Across Study Centers

We developed a simple stratification criterion to categorize patients into two groups: those with missing values in any one of the laboratory parameters and those with complete data for all parameters. Subsequent statistical analyses and comparisons revealed that no significant differences were observed between these two groups with respect to key baseline characteristics (all comparisons yielded P-values > 0.05). These findings strongly indicate that the occurrence of missing data was random rather than associated with specific patient subgroups. (Table 2)

Table 2. Comparison of Baseline Characteristics Between Patients with Missing Data and Those with Complete Data

|  | Zhongnan Hospital of Wuhan University | | Xishui County People's Hospital | |
| --- | --- | --- | --- | --- |
| Variable | Statistic | P-value | Statistic | P-value |
| Age at ART Initiation | -0.41 | 0.682 | -0.109 | 0.913 |
| Delay in ART Initiation | -1.541 | 0.123 | -1.697 | 0.090 |
| Baseline CD4⁺ T Cell Count | -1.194 | 0.233 | -0.246 | 0.806 |
| BMI | -0.64 | 0.522 | -0.199 | 0.842 |
| Sex | - | 1.000 | - | 0.568 |
| HBsAg | - | 0.142 | - | 1.000 |
| Anti-HCV | - | 0.153 | - | 1.000 |
| WHO Clinical Stage | 0.429 | 0.513 | 0.113 | 0.737 |
| ART Regimen | 2.387 | 0.354 | - | 1.000 |
| Route of Infection | 0.645 | 0.682 | 3.21 | 0.167 |

Note: The Mann-Whitney U test was used for the following variables: Age at ART Initiation, Delay in ART Initiation, Baseline CD4⁺ T Cell Count, and BMI. Fisher’s exact test was used for the following variables: Sex, HBsAg, Anti-HCV, WHO Clinical Stage, ART Regimen, and Route of Infection.
